# Prenatal Tobacco Exposure, Brain Subcortical Volumes, and Gray-White Matter Contrast

**DOI:** 10.1001/jamanetworkopen.2024.51786

**Published:** 2024-12-19

**Authors:** Troy B. Puga, Gaelle E. Doucet, Grace E. Thiel, Elijah Theye, Hongying Daisy Dai

**Affiliations:** 1 College of Public Health, University of Nebraska Medical Center, Omaha; 2 College of Osteopathic Medicine, Kansas City University, Kansas City, Missouri; 3 Institute for Human Neuroscience, Boys Town National Research Hospital, Boys Town, Nebraska; 4 Center for Pediatric Brain Health, Boys Town National Research Hospital, Boys Town, Nebraska; 5 Department of Pharmacology and Neuroscience, Creighton University School of Medicine, Omaha, Nebraska

## Abstract

**Importance:**

Maternal tobacco use during pregnancy (MTDP) remains a major public health challenge. However, the complete spectrum of effects of MTDP is not fully understood.

**Objectives:**

To examine the longitudinal associations of MTDP and children’s brain morphometric subcortical volume and gray-white matter contrast (GWC) development.

**Design, Setting, and Participants:**

Cohort study of children aged 9 to 10 years at wave 1 (October 2016 to October 2018) and at a 2-year follow-up (wave 2; August 2018 to January 2021; aged 11-12 years) across 21 US sites in the Adolescent Brain Cognitive Development (ABCD) Study. Data were analyzed from October 2023 to October 2024.

**Exposure:**

MTDP.

**Main outcomes and measures:**

Morphometric brain measures of subcortical volume and GWC.

**Results:**

Among the 11 448 children (51.5% male; 13.1% Black; 24.0% Hispanic; and 52.9% White) at wave 1, 1607 (16.6%; 95% CI, 13.0%-20.2%) were identified with MTDP exposure. At wave 1, children with MTDP exposure (vs no exposure) exhibited lower GWC in widespread brain regions primarily located in the frontal (eg, superior frontal; regression coefficient [*B*] = −0.0019; SE, 0.0006; *P* = .004), parietal (eg, supramarginal; *B* = −0.0021; SE, 0.0007; *P* = .002) and temporal lobes (eg, middle temporal; *B* = −0.0024; SE, 0.0007; *P* < .001). These differences in GWC continued to be significant at wave 2. In regard to subcortical volume, children with MTDP exposure demonstrated smaller volume of the lateral ventricle (*B* = −257.5; SE, 78.6; *P* = .001) and caudate (*B* = −37.7; SE, 14.0; *P* = .01) in the left hemisphere at wave 1, and lower volume of the caudate in both left (*B* = −48.7; SE, 15.9; *P* = .002) and right hemisphere (*B* = −45.5; SE, 16.1; *P* = .01) at wave 2.

**Conclusions and Relevance:**

This cohort study found that MTDP exposure was associated with lower GWC across the whole cortex and smaller caudate nuclei volume compared with no exposure, signifying the importance of preventing MTDP and necessitating further research on this topic.

## Key Points

**Question:**

Does exposure to maternal tobacco use during pregnancy (MTDP) exhibit longitudinal brain morphometric developmental associations in subcortical volume and gray-white matter contrast of offspring in late childhood (aged 9-12 years)?

**Results:**

In this cohort study of 11 448 participants, adolescents exposed to MTDP, compared with those unexposed, had smaller caudate nucleus volumes and lower gray-white matter contrast across widespread regions of the frontal, parietal, and temporal lobes—regions that support higher-order cognitive processing.

**Meaning:**

These findings suggest MTDP exposure had negative associations with childhood brain structures involved with key cognitive functions, which further signifies the need for interventions to prevent MTDP.

## Teaser

This cohort study evaluates longitudinal associations of maternal tobacco use during pregnancy and children’s brain morphometric subcortical volume and gray-white matter contrast development.

## Introduction

Maternal tobacco use during pregnancy (MTDP) remains a prominent public health and global health challenge, with rates still relatively high throughout the world.^1,2^ The prevalence of maternal tobacco use varies widely between regions, with the European region and the region of the Americas having the highest prevalence at 8.1% and 5.9%, respectively.^1^ An expanding body of research highlights the immediate adverse effects of MTDP on maternal health, fetal growth, and newborn development, including higher risks of miscarriage, stillbirth, and low birth weight. Babies born to mothers who smoke are also more prone to preterm birth, respiratory issues such as asthma and infections, and have an increased risk of sudden infant death syndrome.^3^

In a prior study from our group assessing children aged 9 to 12 years, MTDP exposure was associated with adverse neurocognitive effects in children in regard to language processing, crystallized cognition, and episodic memory.^4^ At the brain structural level, MTDP exposure has been associated with various deleterious findings, including lower global and regional brain volumes and suboptimal cortical traits.^4,5^ A recent Generation R Study conducted on 2704 children aged 10 years^5^ found overall smaller cerebral gray and white matter volumes but also a mixture of both bigger and smaller subcortical regions, thicker cortical regions, and smaller surface areas in widespread regions in children exposed to MTDP, compared with unexposed children. Other studies have described that intrauterine exposure to tobacco alters the development and subsequent size of gross brain structures, such as cortical thinning in children aged 6 to 8 years^6^ and adolescents aged between 12 and 18 years.^7^ A study done in South Africa showed that MTDP exposure led to smaller volumes in the lateral orbitofrontal cortex and nucleus accumbens as well as smaller surface area in several brain regions primarily located in the frontal cortex in children aged 8 to 11 years.^8,9^ At the brain functional level, studies have reported that MTDP exposure was associated with weaker activation in frontal and parietal regions during an inhibitory control task in young adults.^10^ Overall, these findings may reflect suboptimal brain development in children and adolescents whose mothers used tobacco during pregnancy.^11^ Gray-white matter contrast (GWC) has been suggested to be a marker of intracranial myelin, which is sensitive to maturational changes during brain development and has been related to cognitive functions,^12,13,14^ but also sensitive to neurodevelopmental and neuropsychiatric disorders.^15,16,17,18^ Given that MTDP exposure has been associated with increased risk for psychiatric comorbidities,^19^ such metrics may be informative to further explore the potential effects of exposure to MTDP regarding offspring’s brain development during adolescence.

Nicotine has been shown to have many negative effects on childhood brain development, such as impacting cortical thickness and gene expression.^6,20,21^ Nicotine use in early childhood has been shown to have negative effects on child language processing.^21^ Yet, the full spectrum of effects of exposure to MTDP remains limited, and further research is necessary to understand these effects. Following our previous works,^4^ this study further analyzed data from the Adolescent Brain Cognitive Development (ABCD) Study to examine the longitudinal associations between MTDP exposure and the development of subcortical brain volume and GWC in children aged 9 to 10 and 11 to 12 years. Based upon the prior evidence of MTDP exposure,^5^ we hypothesized that children whose mothers used tobacco during pregnancy would show differences in subcortical volumes, including larger amygdala and putamen and smaller caudate and nucleus accumbens. Furthermore, based on animal studies^22^ showing that cigarette smoke exposure has been linked to reduced myelin synthesis and maintenance and our previous work suggesting lower regional surface area and cortical volumes, we expected an overall lower GWC in children with MTDP exposure than children without exposure.^4^

## Methods

### Participants and Data Acquisition

This cohort study used data from the ABCD Study Release 4.0, which is located within the National Data Archive. Children aged 9 to 10 years were enrolled at baseline between October 2016 and October 2018 from 21 sites across the US (wave 1).^23^ Participants were recruited using school-based probability sampling, which accounted for factors such as socioeconomic position, self-reported race and ethnicity (Asian, Black, Hispanic, White, and other [American Indian or Alaska Native, Native Hawaiian or Other Pacific Islander, other races, or multiracial groups]), and sex assigned at birth.^24^ Race and ethnicity were assessed as social constructs. Parents or guardians and the children involved were informed of the study’s purpose, procedures, potential risks, and benefits, after which parents or legal guardians provided written consent, and child participants offered assent to participate in the study. Participants underwent a comprehensive evaluation including surveys, cognitive testing, and neuroimaging assessment at baseline.^24,25,26^ Follow-up cognitive performance and neuroimaging testing were completed from August 2018 to January 2021 (wave 2). The original ABCD study was approved by the institutional review board of the University of California, San Diego. This study adheres to the Strengthening the Reporting of Observational Studies in Epidemiology (STROBE) reporting guideline.

### Inclusion and Exclusion Criteria

The ABCD study included 11 876 participants at wave 1 and 10 414 participants at wave 2. After excluding those with missing MTDP exposure status and missing Freesurfer measurements, 11 448 participants in wave 1 and 9846 participants in wave 2 were eligible for participation in our study. Participants were then excluded if they had missing magnetic resonance imaging (MRI), poor neuroimaging, and concerning medical factors outside of MTDP, or subsequent health-related issues. Details of the sample selection process can be found in the eAppendix in Supplement 1.^4^

### Maternal Tobacco Use During Pregnancy

Exposure to medications, drugs, alcohol, and tobacco during the prenatal period was assessed through clinical evaluation and a parental survey of medical history, which included 2 questions: “Did you use tobacco before knowing about the pregnancy?” and “Did you use tobacco after knowing about the pregnancy?” Children whose mothers provided a yes response to prenatal tobacco exposure, either before or after awareness of pregnancy, were classified into the MTDP group, with the remainder serving as the control group of no MTDP exposure.

### Covariates

For child participants, factors such as age, sex assigned at birth, self-reported race and ethnicity as social constructs, tobacco use, substance use, pubertal stage, intracranial volume, parental monitoring, school environment, handedness, imaging device manufacturer, and study site were selected to capture a range of variables covering individual, family, and school domains that are known to influence the brain morphometric development based on a review of the existing literature and peer-reviewed analytical protocols.^4,14,21,27^ In the timeline flow-back survey at wave 1, children were asked if they had heard of tobacco products such as cigarettes, smokeless tobacco, cigars, hookah, electronic cigarettes, or e-cigarettes. Those who answered yes were subsequently asked if they had ever tried any tobacco products, with separate questions for each type (e-cigarette, cigarette, cigar, smokeless tobacco, hookah, pipe, and nicotine replacement). Participants who answered yes to any of these questions were classified as ever tobacco users. Ever use of other substance was self-reported by participants for alcohol use, marijuana use, and the use of any other known drugs (eg, cocaine, methamphetamine, MDMA, inhalants, stimulants, anabolic steroids, sedatives, opioids, and over-the-counter cold medicine).

Children self-rated puberty development using a scale composed of questions regarding development levels of height, body hair, skin, voice, and facial hair (males) or thelarche and menarche (females). A higher score on the self-rated puberty development scale indicated an increased level of pubertal development. The child-reported parent monitoring scale comprised 5 questions about perceived parental monitoring. Higher scores on the perceived parental monitoring scale signaled higher levels of parental monitoring.^28^ The school risk environment survey asked 6 questions about school and environmental risk.^29^ Higher scores correlated with a better school environment.

### Statistical Analysis

Summary statistics for sample characters and mean (SE) of GWC and subcortical volume were reported in wave 1 (baseline) and wave 2 (2-year follow-up). Following the statistical guidelines for the ABCD study, we used a weighted approach for inference at the population level. This method accounted for participant clustering across 21 study sites, sample selection biases, and nonresponsiveness in the observational study design. The weighted ABCD data mirrored the demographic composition of the American Community Survey’s third- and fourth-grade enrollment statistics at each site.^24,30^

Multivariable regressions were employed using the linear mixed models to build the variance-covariance matrix for individuals and to estimate sampling errors, taking the study site clustering and sampling weight into account. Primary analyses examined the association between MTDP exposure and brain morphometric measures, adjusting for various covariates.

The neuroimaging analysis focused on associations between MTDP exposure and intensity measures of GWC for 34 regions of interest (ROI) in the frontal, parietal, temporal, and occipital lobes, as well as 18 subcortical volumes, which were analyzed separately. The regression models for morphometric measures also included factors such as handedness (left vs right) and MRI device manufacturer. Adjusted regression coefficients (*B*) and SEs were calculated. Statistical analyses were performed using SAS version 9.4 (SAS Institute). Due to the low incidence of missing data, a complete case analysis was conducted. To control the study-wise false discovery rate (FDR) at a 2-sided .05 significance level, a Benjamini-Hochberg correction was applied.^31^ Data were analyzed from October 2023 to October 2024. To investigate the group differences observed in GWC, we conducted a detailed analysis of gray and white matter intensity, applying the same statistical approach.

## Results

A total of 9991 participants in wave 1 and 6721 participants in wave 2 were included in the final analyses. The sample comprised a diverse youth population, including 51.5% (95% CI, 50.5%-52.4%) male children, 13.1% (95% CI, 7.9%-18.4%) Black children, 24.0% (95% CI, 11.0%-37.0%) Hispanic children, and 52.9% (95% CI, 40.9%-64.8%) White children. The mean (SD) of the Puberty Development Scale (range: 1-4), School Risk Environment Survey (range: 6-24), and Parent Monitoring Scale (range: 1-5) were 1.8 (0.6), 19.9 (2.8), and 4.4 (0.5), respectively. Wave 2 included 9846 children aged 11 to 12 years, with 1308 (15.8%) having been identified with MTDP exposure. Sample characteristics were similar between the 2 waves. Descriptive statistics (mean and standard deviation) and the associations between covariates and MTDP exposure can be found in our previous work.^4^ Briefly, MTDP was more pronounced among Black (vs White) adolescents and children with lower (vs higher) parental education and family income. The comparison in eTable 1 in Supplement 1 shows no significant difference in MTDP exposure between participants who completed both waves and those lost to follow-up (Table 1). Among 11 448 children aged 9 and 10 years at wave 1, 1607 (16.6%) were identified with MTDP exposure.

**Table 1.** Characteristics of Offspring Participants

Characteristic	No. (Weighted %) [95% CI]^a^	
	Wave 1, aged 9-10 y (n = 11 448)	Wave 2, aged 11-12 y (n = 9846)
Race and ethnicity		
Asian	221 (3.3) [1.4-5.1]	180 (3.1 (1.2-4.9]
Black	1668 (13.1) [7.9-18.4]	1288 (11.9) [6.8-16.9]
Hispanic	2327 (24.0) [11.0-37.0]	1989 (23.9) [10.9-36.9]
White	6040 (52.9) [40.9-64.8]	5373 (54.5) [42.3-66.7]
Other^b^	1190 (6.7) [4.6-8.9]	1016 (6.7) [4.4-9.0]
Sex	6040 (52.9) [40.9-64.8]	5373 (54.5) [42.3-66.7]
Male	5990 (51.5) [50.5-52.4]	5161 (51.6) [50.5-52.7]
Female	5458 (48.5) [47.6-49.5]	4685 (48.4) [47.3-49.5]
Maternal tobacco use during pregnancy		
No	9841 (83.4) [79.8-87]	8538 (84.2) [80.5-87.9]
Yes	1607 (16.6) [13-20.2]	1308 (15.8) [12.1-19.5]
Child characteristics, mean (SD) [range]		
Pubertal stage	1.8 (0.6) [1.0-4.0]	1.8 (0.6) [1.0-4.0]
School environment	19.9 (2.8) [6.0-24.0]	19.9 (2.8) [6.0-24.0]
Parent monitoring	4.4 (0.5) [1.0-5.0]	4.4 (0.5) [1.6-5.0]
Whole brain measures, mean (SD) [range]		
Total cortical surface area, mm^2^	189 711.0 (18 177.6) [128 087.0-275 078.0]	190 384.3 (18 216.9) [124 895.0-270 709.0]
Mean cortical thickness, mm	2.7 (0.1) [2.3-3.1]	2.7 (0.1) [2.2-3.0]
Total cortical volume, mm^3^	597 253.7 (56 182.4) [394 059.0-832 508.0]	588 735.0 (57 002.0) [370 573.0-815 808.0]
Subcortical gray matter volume, mm^3^	59 965.9 (4884.6) [30 981.0-81 185.0]	60 773.9 (4960.6) [37 624.0-81 469.0]
Cerebral white matter volume, mm^3^	420 493.2 (48701.1) [264 715.0-684 355.0]	435 018.2 (50 125.6) [257 639.0-703 213.0]
Intracranial volume, mm^3^	1 490 716.1 (143 524.8) [983 742.6-2 078 560.4]	1 522 432.2 (147 526.6) [993 777.4-2 155 652.8]

^a^ Weighted statistics, which account for sampling weights and site clustering, are reported for population-level inference.

^b^ Other included individuals who identified as American Indian or Alaska Native, Native Hawaiian or other Pacific Islander, other races, or multiracial groups.

In both waves, participants with MTDP exposure showed lower GWC in widespread regions located throughout the frontal, parietal, and temporal lobes, compared with the participants without MTDP exposure (Table 2, Figure). In detail, participants with MTDP exposure had lower GWC in the following regions in the temporal lobe: superior temporal at wave 1 (*B* = −0.0023; SE, 0.0006; *P* < .001) and wave 2 (*B* = −0.0028; SE, 0.0007; *P* < .001), inferior temporal at wave 1 (*B* = −0.0018; SE, 0.0006; *P* = .003) and wave 2 (*B* = −0.0027; SE, 0.0007; *P* < .001), middle temporal at wave 1 (*B* = −0.0024; SE, 0.0007; *P* < .001) and wave 2 (*B* = −0.0029; SE, 0.0008; *P* < .001), banks of superior temporal sulcus at wave 1 (*B* = −0.0024; SE, 0.0007; *P* = .001) and wave 2 (*B* = −0.0026; SE, 0.0008; *P* = .002), fusiform gyrus at wave 1 (*B* = −0.0019; SE, 0.0006; *P* = .001) and wave 2 (*B* = −0.0022; SE, 0.0006; *P* < .001), entorhinal gyrus at wave 1 (*B* = −0.003; SE, 0.0009; *P* = .001) and wave 2 (*B* = −0.0031; SE, 0.001; *P* = .003), and parietal (eg, supramarginal; *B* = −0.0021; SE, 0.0007; *P* = .002) at wave 1. See Table 2 for additional significant regions in other lobes.

**Table 2.** Region of Interest Analysis of Gray-White Matter Contrast (GWC) Integrity Measures

GWC^a^	Wave 1 (aged 9-10 y)					Wave 2 (aged 11-12 y)				
	Participants by MTDP, weighted mean (SE)		Adjusted *B* (SE)^b^	Adjusted *P* value	FDR-corrected *P* value^c^	Participants by MTDP, weighted mean (SE)		Adjusted *B* (SE)^b^	Adjusted *P* value	FDR-corrected *P* value^c^
	No (n = 8634)	Yes (n = 1357)				No (n = 5812)	Yes(n = 909)			
Frontal										
Superior frontal	0.26 (0.004)	0.25 (0.004)	−0.0019 (0.0006)	.004	.01	0.25 (0.004)	0.25 (0.004)	−0.003 (0.0007)	<.001	<.001
Rostral middle	0.28 (0.004)	0.28 (0.004)	−0.0015 (0.0007)	.04	.06	0.28 (0.004)	0.27 (0.005)	−0.0031 (0.0008)	<.001	<.001
Caudal middle	0.26 (0.004)	0.26 (0.003)	−0.0027 (0.0007)	<.001	.002	0.26 (0.004)	0.25 (0.004)	−0.0036 (0.0008)	<.001	<.001
Pars opercularis	0.27 (0.005)	0.27 (0.006)	−0.0028 (0.0006)	<.001	<.001	0.27 (0.006)	0.27 (0.007)	−0.0028 (0.0007)	<.001	<.001
Pars triangularis	0.27 (0.005)	0.27 (0.005)	−0.0019 (0.0007)	.01	.01	0.27 (0.005)	0.27 (0.006)	−0.0032 (0.0008)	<.001	<.001
Pars orbitalis	0.26 (0.004)	0.26 (0.005)	−0.0017 (0.0007)	.02	.05	0.26 (0.005)	0.26 (0.006)	−0.0031 (0.0009)	<.001	.001
Lateral orbitofrontal	0.26 (0.005)	0.25 (0.006)	−0.0019 (0.0006)	.002	.01	0.26 (0.006)	0.25 (0.007)	−0.0021 (0.0007)	.002	.01
Medial orbitofrontal	0.26 (0.004)	0.26 (0.005)	−0.0008 (0.0007)	.27	.34	0.26 (0.005)	0.26 (0.007)	−0.0019 (0.0009)	.04	.05
Precentral	0.23 (0.004)	0.22 (0.003)	−0.002 (0.0006)	.001	.004	0.22 (0.003)	0.21 (0.003)	−0.0023 (0.0007)	<.001	.002
Paracentral	0.23 (0.004)	0.23 (0.004)	−0.0013 (0.0006)	.04	.06	0.22 (0.004)	0.22 (0.005)	−0.002 (0.0007)	.004	.01
Frontal pole	0.25 (0.003)	0.24 (0.003)	−0.002 (0.001)	.04	.06	0.25 (0.003)	0.24 (0.004)	−0.0033 (0.0011)	.004	.01
Rostral anterior	0.25 (0.005)	0.25 (0.006)	0 (0.0007)	.97	.99	0.25 (0.005)	0.25 (0.007)	−0.0016 (0.0008)	.03	.05
Caudal anterior	0.3 (0.006)	0.3 (0.007)	−0.0005 (0.0008)	.51	.58	0.3 (0.007)	0.3 (0.009)	−0.0015 (0.001)	.12	.15
Parietal										
Superior parietal	0.25 (0.003)	0.25 (0.003)	−0.0013 (0.0007)	.05	.07	0.24 (0.003)	0.24 (0.004)	−0.0017 (0.0008)	.02	.04
Inferior parietal	0.26 (0.004)	0.26 (0.005)	−0.0013 (0.0007)	.06	.09	0.26 (0.004)	0.26 (0.006)	−0.002 (0.0008)	.01	.02
Supramarginal	0.27 (0.004)	0.26 (0.004)	−0.0021 (0.0007)	.002	.01	0.26 (0.004)	0.26 (0.005)	−0.0023 (0.0007)	.002	.01
Postcentral	0.22 (0.003)	0.22 (0.003)	−0.0008 (0.0006)	.15	.21	0.22 (0.003)	0.21 (0.003)	−0.0014 (0.0006)	.02	.03
Precuneus	0.26 (0.005)	0.26 (0.006)	−0.0015 (0.0006)	.02	.04	0.26 (0.005)	0.25 (0.007)	−0.0021 (0.0007)	.003	.01
Posterior cingulate	0.28 (0.006)	0.28 (0.006)	−0.0014 (0.0007)	.04	.06	0.28 (0.006)	0.28 (0.008)	−0.0014 (0.0007)	.04	.06
Isthmus cingulate	0.25 (0.005)	0.25 (0.006)	−0.0016 (0.0006)	.004	.01	0.25 (0.006)	0.25 (0.007)	−0.0018 (0.0006)	.01	.01
Temporal										
Superior temporal	0.26 (0.005)	0.26 (0.005)	−0.0023 (0.0006)	<.001	.001	0.26 (0.005)	0.25 (0.006)	−0.0028 (0.0007)	<.001	<.001
Inferior temporal	0.27 (0.005)	0.26 (0.005)	−0.0018 (0.0006)	.003	.01	0.26 (0.005)	0.26 (0.006)	−0.0027 (0.0007)	<.001	.001
Middle temporal	0.27 (0.005)	0.27 (0.004)	−0.0024 (0.0007)	<.001	.002	0.27 (0.005)	0.26 (0.005)	−0.0029 (0.0008)	<.001	.001
Banks of superior temporal sulcus	0.3 (0.006)	0.29 (0.007)	−0.0024 (0.0007)	.001	.004	0.29 (0.006)	0.29 (0.008)	−0.0026 (0.0008)	.002	.01
Fusiform	0.26 (0.006)	0.25 (0.007)	−0.0019 (0.0006)	.001	.004	0.25 (0.006)	0.25 (0.008)	−0.0022 (0.0006)	<.001	.001
Transverse temporal	0.2 (0.004)	0.2 (0.005)	0 (0.0006)	.99	.99	0.19 (0.005)	0.19 (0.006)	−0.0004 (0.0007)	.57	.63
Entorhinal	0.22 (0.006)	0.21 (0.008)	−0.003 (0.0009)	.001	.004	0.22 (0.006)	0.22 (0.009)	−0.0031 (0.001)	.003	.01
Temporal pole	0.22 (0.005)	0.22 (0.005)	−0.001 (0.0008)	.21	.28	0.22 (0.005)	0.22 (0.006)	−0.0017 (0.0009)	.06	.07
Parahippocampal	0.24 (0.006)	0.24 (0.008)	0.0003 (0.0007)	.71	.78	0.24 (0.006)	0.24 (0.009)	−0.0007 (0.0008)	.40	.45
Occipital										
Lateral occipital	0.22 (0.003)	0.22 (0.004)	−0.0001 (0.0006)	.88	.93	0.21 (0.003)	0.21 (0.004)	−0.0008 (0.0006)	.23	.27
Lingual	0.21 (0.005)	0.21 (0.006)	0.001 (0.0005)	.04	.06	0.2 (0.005)	0.2 (0.007)	0.0002 (0.0005)	.77	.79
Cuneus	0.2 (0.004)	0.2 (0.005)	0.0007 (0.0005)	.19	.26	0.19 (0.004)	0.19 (0.006)	0 (0.0006)	.96	.96
Pericalcarine	0.18 (0.004)	0.18 (0.005)	0.0004 (0.0005)	.40	.47	0.17 (0.005)	0.17 (0.006)	0.0002 (0.0006)	.77	.79
Insula cortex										
Insula	0.19 (0.005)	0.19 (0.005)	−0.0005 (0.0004)	.30	.36	0.19 (0.005)	0.19 (0.006)	−0.001 (0.0005)	.04	.06

Abbreviations: *B*, regression coefficient; FDR, false discovery rate; MTDP, maternal tobacco use during pregnancy.

^a^ Weighted mean and SE of GWC measures, defined as (white matter – gray matter) / ([white matter + gray matter] / 2) of T1 weighted image for aparc regions of interest.

^b^ The dependent variables were GWC in 34 regions of interest. The independent variable was MTDP exposure. The analysis was adjusted by covariates, including age, sex, race and ethnicity, intracranial volume, pubertal stage, substance ever use, tobacco ever use, parental monitoring, school environment, handedness, imaging device manufacturer, and study site. Sampling weights were incorporated to remove the sampling bias. *B* measured the difference in GWC variables by MTDP.

^c^ False discovery rate (FDR) correction was performed across 34 regions to prevent inflation of type I errors.

**Figure.**
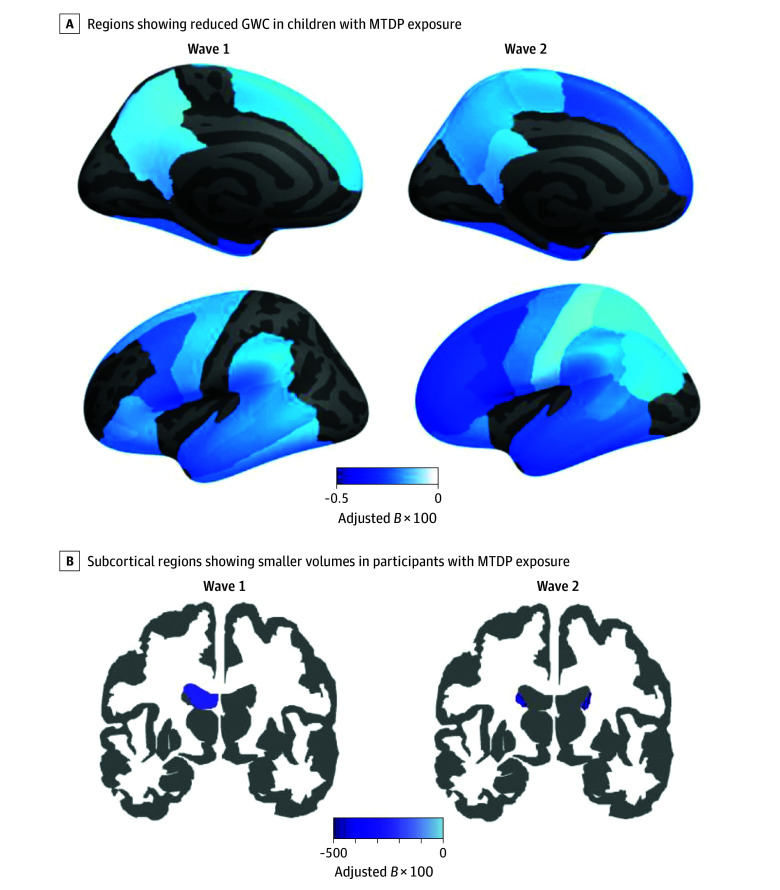
Display of the Significant Regions Between Participants With Maternal Tobacco Use During Pregnancy (MTDP) and Without MTDP at Each Wave A, Regions showing reduced GWC in children with MTDP exposure. Left hemisphere is displayed. Darker blue reflects more severe reduction. B, Subcortical regions showing smaller volumes in participants with MTDP exposure. Only regions with significance at false discovery rate are shown. *B* indicates regression coefficient; GWC, gray-white matter contrast.

As shown in Table 3, regarding the subcortical volumes, participants with MTDP exposure demonstrated smaller left ventricle (*B* = −257.5; SE, 78.6; *P* = .001) and caudate nuclei (left: *B* = −37.7; SE, 14.0; *P* = .01) in wave 1, and caudate nuclei (right: *B* = −45.5; SE, 16.1; *P* = .01; left: *B* = −48.7; SE, 15.9; *P* = .002) in wave 2, relative to participants without MTDP exposure (Table 3, Figure, B). There was statistically higher gray matter intensity in several brain regions (eTable 2 in Supplement 1) with no consistent differences in white matter intensity (eTable 3 in Supplement 1) in children exposed to MTDP compared with the unexposed children.

**Table 3.** Longitudinal Comparison of Subcortical Volume Among Children by Maternal Tobacco Use During Pregnancy (MTDP)^a^

Subcortical volume	Wave 1, aged 9-10 y				Wave 2, aged 11-12 y			
	Participants by MTDP, weighted mean (SE), mm^3^		Adjusted *B* (SE)^a^^,^^b^	*P* value^a^^,^^c^	Participants by MTDP, weighted mean (SE), mm^3^		Adjusted *B* (SE)^a^^,^^b^	*P* value^a^^,^^c^
	No (n = 8634)	Yes (n = 1357)			No (n = 5812)	Yes (n = 909)		
Left hemisphere								
Lateral ventricle	5175.4 (47.4)	4877.0 (62)	−257.5 (78.6)	.001	5524.5 (67.1)	5256.8 (106.9)	−247.8 (101.9)	.02
Cerebellum cortex	57 082.0 (315.6)	56 600.0 (289.0)	−347.8 (150.5)	.02	57 740.0 (321.7)	56 957.0 (273.3)	−402.2 (180.5)	.03
Thalamus	7789.3 (33.3)	7710.2 (28.5)	−26.9 (19.7)	.17	7962.5 (39.8)	7834.7 (31.1)	−37.9 (25.1)	.13
Caudate	3865.1 (14.5)	3792.7 (17.6)	−37.7 (14)	.01	3868.1 (16.4)	3772.6 (14.5)	−48.7 (15.9)	.002
Putamen	5307.1 (13.8)	5282.9 (20.0)	22.0 (17.5)	.21	5332.2 (17.6)	5280.9 (23.5)	17.2 (20.5)	.40
Pallidum	1994.7 (15.2)	1980.7 (14.8)	1.6 (7.3)	.83	2015.5 (19.2)	1988.8 (19.9)	−2.2 (8.3)	.79
Hippocampus	4016.9 (15.5)	3969.0 (12.1)	−19.3 (10.5)	.07	4082.1 (18.0)	4010.3 (16.0)	−24.8 (13.4)	.06
Amygdala	1716.0 (20.3)	1691.9 (22.6)	−6.4 (6.4)	.32	1743.2 (23.8)	1706.4 (27.9)	−13.2 (7.2)	.07
Accumbens area	589.1 (5.5)	575.0 (4.6)	−4.7 (3)	.12	587.0 (6.3)	568.3 (4.8)	−3.9 (3.7)	.29
Right hemisphere								
Lateral ventricle	4731.4 (46.5)	4627.3 (49.7)	−83.6 (82.4)	.31	5052.3 (63.5)	4983.9 (87.4)	−92.5 (109.3)	.40
Cerebellum cortex	57781.0 (327.0)	57 332.0 (292.5)	−332.2 (153.1)	.03	58 571.0 (335.5)	57 836.0 (305.4)	−429.8 (189.6)	.02
Thalamus	7314.6 (28.5)	7257.7 (27.3)	−13.3 (17.5)	.45	7439.0 (34.7)	7343.4 (30.3)	−33.2 (21.5)	.12
Caudate	4025.2 (14.6)	3960.6 (16.1)	−30.7 (14)	.03	4016.8 (16.4)	3927.0 (12.6)	−45.5 (16.1)	.01
Putamen	5408.4 (15.3)	5380.7 (20.6)	18.2 (16.7)	.28	5419.7 (21.7)	5359.6 (21.9)	5.6 (20.6)	.78
Pallidum	1901.7 (8.4)	1882.5 (7.6)	−1.8 (6.8)	.79	1943.8 (8.0)	1924.4 (7.9)	1.8 (8.1)	.82
Hippocampus	4146.5 (16.3)	4090.4 (15.6)	−16.6 (11.2)	.14	4218.8 (20.2)	4140.3 (21.1)	−30.7 (13.7)	.03
Amygdala	1830.2 (8.6)	1806.4 (7.2)	−7.8 (5.9)	.19	1860.5 (9.1)	1829.5 (7.1)	−7.3 (6.9)	.29
Accumbens area	622.9 (2.9)	609.3 (3.3)	−6 (2.9)	.04	616.6 (3.2)	598.6 (3.7)	−5.2 (3.5)	.13

Abbreviation: *B*, regression coefficient.

^a^ Multivariate regression analyses were performed where the dependent variables were subcortical volume. The independent variable was MTDP. The analysis was adjusted by covariates, including age, sex, race and ethnicity, intracranial volume, pubertal stage, substance ever use, tobacco ever use, parental monitoring, school environment, handedness, imaging device manufacturer, and study site. Sampling weights were incorporated to remove the sampling bias.

^b^ *B* measured the difference in subcortical volume by MTDP exposure.

^c^ False discovery rate (FDR) correction was performed to prevent inflation of type I errors.

## Discussion

This cohort study showcased the associations between MTDP exposure and 2 brain morphometric variables—GWC and subcortical volumes—in children aged 9 to 12 years, with consistent findings throughout the 2 waves. In this sample of typically developing children, the lower subcortical volume may signify a higher risk for neurological deterioration and cognitive impairments, whereas GWC changes may indicate altered neuronal maturation with neuronal damage or microstructural changes. Cortical and subcortical structures are known to be influenced by genetics^32^ and are highly sensitive to brain development^12^ and psychopathology.^17^

One of our main findings revealed that children with MTDP exposure had significantly lower GWC across widespread regions located in the frontal, parietal, and temporal cortices compared with those without MTDP exposure. GWC has been specifically chosen because it reflects microstructural changes in gray and white matter, including pericortical myelin content, which has been further found to significantly influence cortical thickness.^33,34^ Animal studies have also demonstrated that myelin synthesis and maturation are sensitive to smoke exposure.^22^ Furthermore, this metric has been shown to have unique genetic influences^32^; be sensitive to age throughout adulthood^33,35^ and early development,^12,36^ as well as psychopathology^17^; and be a potential marker of cognitive changes.^12,13^ All our results showed a consistent reduction in cortical GWC in children with MTDP exposure. The underlying mechanism behind such reduction is not well understood. It has been suggested to be caused by either a reduction in white matter signal intensity, which may reflect myelin structural changes,^37^ or an increase in gray matter signal intensity, which has been generally linked to changes in water content^34^ or neuronal loss.^38^

Given the large dataset offered by the ABCD project, we were further able to investigate both distinctions (ie, changes in white matter vs gray matter intensity) separately in the eAppendix analyses in Supplement 1, showing higher gray matter intensity in some brain regions, while no differences in white matter intensity were associated with MDTP. Therefore, we conjecture that the reductions in GWC revealed in the current study are more likely due to an increased gray matter signal resulting from decreased water content rather than a decreased white matter signal due to demyelination. Water content is also highly correlated with iron content^39^; but it is not possible to determine the exact origin of the change in gray matter intensity. Reduced water content has been shown to be sensitive to age, especially in the infant stage^40,41^ and has been described in several neurological and neuropsychiatric disorders in adult populations.^42,43^ While reductions in regional GWC have been reported throughout development,^40^ and brain regional myelin reduction has been linked with gestational nicotine exposure in animal models,^44^ to our knowledge, no one has yet attempted to determine the underlying mechanisms behind the change in gray matter intensity or GWC in adolescents and whether it is linked to a change in intracortical myelin or water content. Further studies will be essential to understanding these mechanisms.

Most of the differences in GWC found in children with MTDP exposure have been located in the frontal, temporal, and parietal lobes, consistently in both waves. These regions largely encompass the associative cortex, which supports higher-order cognitive functions, such as executive function, language, or memory. Such findings are in line with our previous research, which has shown that children whose mothers used tobacco during pregnancy have been associated with language processing challenges, lower crystallized cognition, and lower episodic memory.^4^

Our analyses on subcortical volumes revealed consistently smaller bilateral caudate nuclei in both left and right hemispheres across both waves, which is consistent with previous studies.^5,45^ The caudate nucleus plays a critical role in various cognitive functions, such as planning the execution of movement, learning, memory, reward, motivation, and emotional processing.^46^ It has also been described as having abnormal activity across several neuropsychiatric disorders such as depressive disorder^47^ or schizophrenia.^48^ Lastly, it should also be noted that at an uncorrected threshold, we also found smaller volumes of the cerebellum and of the right accumbens area, which have also been associated with children being exposed to prenatal smoking in clinical studies.^19,45,49^ In this context, our results suggest that MDTP exposure could be a potential factor associated with risk for neuropsychiatric disorders, and it will be essential to follow up with the future waves from the ABCD sample to determine whether this is the case or not.

This study has highlighted several widespread brain areas negatively associated with MTDP exposure. These areas are located throughout the associative cortices, which are typically supporting all major higher-order cognitive functions. These findings come in addition to the previously recognized negative impacts of MTDP exposure on episodic memory and language processing.^4^ Together, our work shows that the spectrum of associations on offspring whose mothers use tobacco during pregnancy might be much more expansive than previously thought. This information further shows the importance of interventions and public health campaigns to prevent MTDP and to provide assistance to pregnant mothers on smoking cessation.

### Limitations

This study had limitations. First, the analysis of prenatal tobacco exposure did not differentiate between exposure before or after maternal awareness of pregnancy or account for any possible cessation during its course. Research that differentiates cessation before or after awareness of maternal pregnancy has suggested a significant impact on offspring,^5^ and could provide important information for clinicians, as well as possibly aid in the identification of the timing of the greatest impact during the course of pregnancy. Second, separate regression models were used for each region and adjusted for multiple testing. Thus, the region-specific analysis did not account for correlations between regions. While this method is commonly used in the literature,^4,14,21^ future studies should aim to integrate regions and account for interregional correlations. Third, MTDP is self-reported, and it is subject to recall bias. The ABCD study did not include biomarker assessment to quantify the amount of nicotine and exposure to other toxicants during pregnancy, which limits the comprehensive evaluation of nicotine’s role in MTDP on offspring cognitive development. The effects of MTDP likely extend beyond nicotine alone, as other tobacco constituents, such as metals, may also have an impact. Fourth, paternal smoking and other maternal and/or family characteristics during pregnancy (eg, lifestyle) might influence youth cognition development, but these variables were not captured in the ABCD study. Additionally, participants with missing data or poor neuroimaging were excluded. However, the missing rate among covariates was low.

## Conclusions

This study adds to the existing literature by showing large, widespread negative associations of MDTP exposure with regional GWC and subcortical volumes that typically support major higher-order cognitive functions. Collectively, these findings highlight the importance of promoting smoking prevention and cessation among pregnant women to prevent negative impacts on their offspring’s brain development.

## Notes

Supplement 1.
eAppendix.
**eTable 1.** Demographic and Health Characteristics of Participants With Follow-Up vs Lost to Follow-Up **eTable 2. **Regional Analysis of Gray Matter Intensity Measures **eTable 3.** Regional Analysis of White Matter Intensity Measures
eReferences.

Supplement 2.
Data Sharing Statement
