# Supplementary material for: Prenatal Tobacco Exposure, Brain Subcortical Volumes, and Gray-White Matter Contrast
Source: JAMA Netw Open. 2024 Dec 19;7(12):e2451786. doi: 10.1001/jamanetworkopen.2024.51786 (PMC11659912; doi:10.1001/jamanetworkopen.2024.51786)
Supplement: Supplement 1. — eAppendix. eTable 1. Demographic and Health Characteristics of Participants With Follow-Up vs Lost to Follow-Up eTable 2. Regional Analysis of Gray Matter Intensity Measures eTable 3. Regional Analysis of White Matter Intensity Measures eReferences. [file jamanetwopen-e2451786-s001.pdf]

Supplemental Online Content

Puga TB, Doucet GE, Thiel GE, Theye E, Dai H. Prenatal tobacco exposure, brain subcortical volumes, and gray-white matter contrast. *JAMA Netw Open*. 2024;7(12):e2451786. doi:10.1001/jamanetworkopen.2024.51786

eAppendix.
eTable 1. Demographic and Health Characteristics of Participants With Follow-Up vs Lost to Follow-Up
eTable 2. Regional Analysis of Gray Matter Intensity Measures
eTable 3. Regional Analysis of White Matter Intensity Measures
eReferences.

This supplemental material has been provided by the authors to give readers additional information about their work.

© 2025 Puga TB et al. *JAMA Network Open.*

eAppendix.

Brain and Neurodevelopment

Early to mid-childhood is a key period of brain development, as gray and white matter volume and density continue expanding during this period[1,2]. Furthermore, intracortical myelination, as measured by GWC, shows a prolonged developmental trajectory, primarily within prefrontal and other association regions, that may facilitate the dynamic experience-dependent configuration of cognitive systems.[3,4] A child's brain volume also increases most during early childhood and continues to grow through adolescence.[5,6] These periods are, therefore, critical for children's brain development to obtain the necessary neurocognitive functions for everyday life.[6] Disruptions to this critical period could have serious longitudinal effects upon children and their mental health.[7] Another important metric allowing to evaluate typical brain development is the volume of subcortical regions, as smaller subcortical volumes have been linked to neurocognitive decline and deterioration and have been found to be correlated to various cognitive functions and mental health during development.[5,8,9] The growth trajectory of subcortical regions during early life has been reported as mixed,[10] with the hippocampus and amygdala showing increases. At the same time, putamen or caudate were associated with decreases with older age during adolescence.[10,11] In regards to clinical implications, smaller -rather than bigger- subcortical volumes have been linked with higher levels of psychopathology,[8,9,12] although there are exceptions with some neuropsychiatric disorders such as anxiety disorders.[13] Collectively, childhood is a critical period for brain and neurodevelopment in the long term. It is therefore important to determine whether exposure to MTDP impacts the brain development during adolescence.

© 2025 Puga TB et al. *JAMA Network Open.*

MRI Neuroimaging Methods and Quality Control

Magnetic resonance imaging (MRI) was undertaken at both wave 1 and wave 2. To ensure homogeneity between all 21 testing sites, MRI imaging methods were standardized across all testing sites. The imaging protocol utilized three Tesla MRI scanner platforms, using multi-channel, adult-size coils.[14] This protocol decision was based on evidence showing that the brain size of children aged 9-10 is roughly 90% to 95% the size of adult brains, as well as the fact that using custom coil sizes for each age was not logistically feasible and would add significant challenges during analyses. Prior to the scan, the children were desensitized to the scanning environment by undergoing a simulation of the scan, and during the scan a child-friendly movie was played to calm the children. During the scan, real-time motion detection and correction systems were employed to correct for any head movement from the child. The software FreeSurfer, version 5.3.0, was used for image reconstruction. To prevent potential confounders, MRI quality control was conducted, removing participants that had poor neuroimaging. T1 weighted measurements of GWC for 34 regions from the Desikan-Killiany-Tourville (DKT atlas)[15], defined as (white matter intensity – gray matter intensity)/(white matter intensity + gray matter intensity)/2) and 18 subcortical volumetric measures were used for this study for their ability to show correlation in development and cognition.[16,17] GWC were constructed in a way that lower values are indicative of less distinct gray matter /white matter intensities (i.e., less contrast) and higher values of high distinct gray matter/white matter intensities (i.e., more contrast).[17,18]

© 2025 Puga TB et al. *JAMA Network Open.*

Participant Inclusion and Exclusion Criteria

The ABCD study enrolled 11,876 participants at Wave 1 (W1: 2016-2018), with 10,414 participants completing the 2-year follow-up (W2: 2018-2021). After excluding those with missing MTDP exposure status (n=291/W1 and 250/W2) and MRI data (n=256/W1 and 2,838/W2), as well as participants with neuroradiology reports indicating hydrocephalus and herniation (n=432/W1 and 349/W2), individuals with poor MRI image quality (n=45/W1 and 41/W2), inadequate FreeSurfer deconstruction (n=344/W1 and 121/W2), cases of traumatic brain injury with loss of consciousness (n=128/W1 and 115/W2), and those with various medical conditions (n=692/W1 and 622/W2) such as muscular dystrophy, multiple sclerosis, sickle cell anemia, neoplastic disorders, cerebral palsy, diabetes, epilepsy, lead poisoning, and cardiac and kidney disorders, the final analytical sample for MRI neuroimaging outcomes included 9,991 participants in Wave 1 and 6,721 in Wave 2. The selection procedure adhered to protocols established in our previous studies and the MRI quality control guidelines for the ABCD study.[9,19] A flowchart of the sampling selection process and variable definitions has been detailed in our earlier work.[7,20]

© 2025 Puga TB et al. *JAMA Network Open.*

eTable 1. Demographic and Health Characteristics of Participants With Follow-Up vs Lost to Follow-Up

	Follow-Up		Lost to Follow-Up		P-value
Characteristics[a]	n	% (95% CI)	n	% (95% CI)	
MTDP Exposure					
No	5593	86.5(85.7-87.3)	3041	86.2(85.1-87.4)	.71
Yes	872	13.5(12.7-14.3)	485	13.8(12.6-14.9)	
Sex					
Male	3473	53.7(52.5-54.9)	1752	49.7(48-51.3)	<.001
Female	2992	46.3(45.1-47.5)	1774	50.3(48.7-52)	
Race/ethnicity					
Asian	3615	55.9(54.7-57.1)	1643	46.6(45.0-48.3)	<.001
Black	828	12.8(12.0-13.6)	598	17.0(15.7-18.2)	
Hispanic	1259	19.5(18.5-20.4)	811	23.0(21.6-24.4)	
White	111	1.7(1.4-2.0)	83	2.4(1.9-2.9)	
Other[b]	652	10.1(9.4-10.8)	389	11.0(10-12.1)	
Other Substance Ever Use					
No	4973	76.9(75.9-77.9)	2761	78.3(76.9-79.7)	.11
Yes	1492	23.1(22.1-24.1)	765	21.7(20.3-23.1)	
Tobacco Ever Use					
No	6414	99.3(99.0-99.5)	3478	98.7(98.4-99.1)	.01
Yes	48	0.7(0.5-1.0)	45	1.3(0.9-1.6)	
Handedness					
Right-handed	5168	79.9(79-80.9)	2810	79.7(78.4-81)	.29
Left-handed	439	6.8(6.2-7.4)	267	7.6(6.7-8.4)	
Mixed	858	13.3(12.4-14.1)	449	12.7(11.6-13.8)	
Mean (SD)					
School Environment	6453	19.9 (2.8)	3519	20.0 (2.8)	.22
Parent Monitoring	6458	4.4 (0.5)	3519	4.4 (0.5)	.22

[a]: Baseline characteristics of offspring participants are compared between those with follow-up vs. lost to follow-up.
[b]: Individuals identified as American Indian or Alaska Native, Native Hawaiian or other Pacific Islander, other races or multi-racial groups.

© 2025 Puga TB et al. *JAMA Network Open.*

eTable 2. Regional Analysis of Gray Matter Intensity Measures[a]

Gray Matter[a] Weighted mean (SE)		Wave 1 (Age: 9-10)					Wave 2 (Age: 11-12)				
		No Exposure (n=8,634)	MTDP Exposure (n=1,357)	Adjusted B[b]	Adjusted p-value	FDR[c]	No Exposure (n=5,812)	MTDP Exposure (n=909)	Adjusted B[b]	Adjusted p-value	FDR[c]
Frontal	Superior frontal	163.8 (0.9)	164 (0.84)	0.1333 (0.0718)	0.06	0.09	164.5 (0.9)	164.7 (0.9)	0.2562 (0.086)	0.003	**0.01[c]**
	Rostral middle	161.8 (1.04)	162.2 (0.95)	0.2014 (0.0833)	0.02	0.05	162.3 (1.04)	162.8 (1.04)	0.3357 (0.1005)	0.001	**0.007[c]**
	Caudal middle	164.1 (0.94)	164.4 (0.81)	0.2051 (0.0779)	0.008	0.03	165 (0.91)	165.5 (0.79)	0.3188 (0.0968)	0.001	**0.007[c]**
	Pars opercularis	162.8 (1.11)	163.2 (1.11)	0.2326 (0.0724)	0.001	0.01	163.4 (1.14)	163.8 (1.25)	0.2615 (0.0894)	0.003	**0.01**
	Pars triangularis	163.3 (1.01)	163.8 (0.93)	0.1732 (0.081)	0.03	0.06	163.8 (1.03)	164.4 (1.03)	0.3089 (0.0969)	0.001	**0.008[c]**
	Pars orbitalis	162 (0.96)	162.6 (0.95)	0.1907 (0.0927)	0.04	0.06	162.3 (0.99)	162.8 (1.05)	0.3251 (0.109)	0.003	**0.01[c]**
	Lateral orbitofrontal	162.7 (1.15)	163.1 (1.28)	0.2182 (0.0772)	0.005	0.02	162.9 (1.23)	163.1 (1.52)	0.2024 (0.0885)	0.02	**0.05[c]**
	Medial orbitofrontal	160.7 (1.02)	161 (1.17)	0.1953 (0.0872)	0.03	0.06	160.7 (1.1)	161 (1.43)	0.2888 (0.1002)	0.004	**0.013[c]**
	Precentral	169.6 (0.92)	170 (0.81)	0.1567 (0.0741)	0.03	0.06	171.2 (0.9)	171.6 (0.78)	0.1989 (0.0986)	0.04	0.09
	Paracentral	170 (0.98)	170 (1.08)	0.0577 (0.0818)	0.48	0.53	171.4 (1.02)	171.3 (1.26)	0.017 (0.0975)	0.86	0.95
	Frontal pole	162 (0.8)	162.7 (0.74)	0.3056 (0.1316)	0.02	0.06	162.3 (0.84)	163.2 (0.89)	0.6491 (0.1504)	<0.001	**0.001[c]**
	Rostral anterior	158.7 (1.13)	158.8 (1.26)	0.1242 (0.082)	0.13	0.17	158.8 (1.22)	158.9 (1.49)	0.2617 (0.0947)	0.006	0.02
	Caudal anterior	158.1 (1.16)	158.2 (1.28)	0.0675 (0.0856)	0.43	0.49	158.4 (1.26)	158.3 (1.55)	0.1616 (0.0962)	0.09	0.16
Parietal	Superior parietal	166.5 (0.86)	166.8 (0.86)	0.146 (0.0772)	0.06	0.08	167.8 (0.85)	167.9 (0.97)	0.0008 (0.0937)	0.99	0.99
	Inferior parietal	163.4 (1.02)	163.9 (1.04)	0.1643 (0.0753)	0.03	0.06	164.2 (1.04)	164.6 (1.2)	0.1172 (0.0868)	0.18	0.26
	Supramarginal	163.6 (1.02)	164.2 (0.96)	0.2487 (0.0787)	0.002	0.01	164.3 (1.02)	165 (1.03)	0.2178 (0.0928)	0.02	0.04
	Postcentral	169.4 (0.98)	169.7 (0.87)	0.0474 (0.0827)	0.57	0.60	170.6 (0.95)	171 (0.85)	0.0343 (0.1049)	0.74	0.87
	Precuneus	163.9 (1.11)	164.1 (1.29)	0.1475 (0.0671)	0.03	0.06	164.8 (1.17)	164.8 (1.51)	0.0777 (0.0807)	0.34	0.48
	Posterior cingulate	160.7 (1.12)	160.8 (1.24)	0.0708 (0.0709)	0.32	0.37	161.3 (1.19)	161.3 (1.52)	0.1112 (0.0793)	0.16	0.25
	Isthmus cingulate	167.3 (1.11)	167.5 (1.28)	0.1723 (0.0748)	0.02	0.06	168.2 (1.17)	168.3 (1.48)	0.1524 (0.0895)	0.09	0.16
Temporal	Superior temporal	165.9 (1.03)	166.5 (1)	0.2652 (0.0681)	<0.001	**0.002[c]**	166.5 (1.04)	167 (1.08)	0.2996 (0.084)	<0.001	**0.004[c]**
	Inferior temporal	163.5 (0.97)	164.2 (0.92)	0.2525 (0.0814)	0.002	0.01	163.9 (0.96)	164.6 (0.95)	0.2594 (0.0925)	0.005	**0.01[c]**
	Middle temporal	163.5 (1.09)	164.3 (0.88)	0.3098 (0.0794)	<0.001	**0.002[c]**	163.9 (1.06)	164.8 (0.84)	0.3819 (0.0958)	<0.001	**0.001[c]**
	Banks of superior temporal sulcus	162.2 (1.16)	162.7 (1.22)	0.2914 (0.0786)	<0.001	**0.002[c]**	162.9 (1.22)	163.3 (1.43)	0.262 (0.0926)	0.005	**0.01[c]**

© 2025 Puga TB et al. *JAMA Network Open.*

	Fusiform	165.9 (1.21)	166.2 (1.38)	0.1881 (0.0714)	0.008	0.03	166.4 (1.26)	166.6 (1.59)	0.2024 (0.0814)	0.01	0.03
	Transverse temporal	171.1 (1.07)	171.3 (1.15)	0.1035 (0.0857)	0.23	0.28	172 (1.13)	172.1 (1.37)	0.0697 (0.1119)	0.53	0.66
	Entorhinal	171.4 (1.45)	171.7 (1.67)	0.3006 (0.1207)	0.01	0.04	171.2 (1.44)	171.2 (1.86)	0.1199 (0.1344)	0.37	0.51
	Temporal pole	166.1 (1.03)	166.5 (1.13)	0.2448 (0.1125)	0.03	0.06	165.9 (1)	166.2 (1.21)	0.2193 (0.1303)	0.09	0.16
	Parahippocampal	167.2 (1.28)	167.3 (1.5)	0.0062 (0.0824)	0.94	0.94	167.3 (1.31)	167.4 (1.7)	0.0605 (0.1002)	0.55	0.66
Occipital	Lateral occipital	170.5 (0.8)	171 (0.71)	-0.0094 (0.0911)	0.92	0.94	171.5 (0.79)	171.9 (0.73)	-0.0721 (0.1063)	0.50	0.65
	Lingual	169 (1.14)	169.2 (1.32)	0.0909 (0.0699)	0.19	0.24	169.6 (1.21)	169.7 (1.54)	0.0093 (0.082)	0.91	0.96
	Cuneus	168.9 (1.1)	169.1 (1.23)	0.1563 (0.0818)	0.06	0.08	169.7 (1.17)	169.8 (1.45)	0.0306 (0.1034)	0.77	0.87
	Pericalcarine	169.1 (1.12)	169.4 (1.24)	0.1785 (0.0852)	0.04	0.06	169.7 (1.21)	169.8 (1.48)	0.0099 (0.1088)	0.93	0.96
Insula Cortex	Insula	165.2 (1.06)	165.4 (1.15)	0.1326 (0.066)	0.04	0.07	165.5 (1.12)	165.6 (1.35)	0.1155 (0.0813)	0.16	0.25

[a]: Average T1 intensity of gray matter voxels 0.2 mm from the white matter surface for APARC ROI.
[b]: The dependent variables were gray matter intensity measures in 34 regions of interest. The independent variable was MTDP exposure. The analysis was adjusted by covariates, including age, sex, race/ethnicity, intracranial volume, pubertal stage, substance ever use, tobacco ever use, parental monitoring, school environment, handedness, imaging device manufacturer, and study site. Sampling weights were incorporated to remove the sampling bias. Regression coefficients (B) measured the difference in gray matter intensity variables by MTDP exposure.
[c]: FDR correction was performed across 34 regions to prevent inflation of Type I errors. Footnote indicates FDR<0.05 (bold).

© 2025 Puga TB et al. *JAMA Network Open.*

eTable 3. Regional Analysis of White Matter Intensity Measures

White Matter[a] Weighted mean (SE)		Wave 1 (age: 9-10) No Exposure (n=8,634)	Wave 1 MTDP Exposure (n=1,357)	Wave 1 Adjusted B[b]	Wave 1 Adjusted p-value	Wave 1 FDR[c]	Wave 2 (age: 11-12) No Exposure (n=5,812)	Wave 2 MTDP Exposure (n=909)	Wave 2 Adjusted B[b]	Wave 2 Adjusted p-value	Wave 2 FDR[c]
Frontal	Superior frontal	186.2 (0.68)	186.2 (0.62)	-0.0237 (0.051)	0.64	0.81	186.7 (0.68)	186.8 (0.66)	0.013 (0.0638)	0.84	0.95
	Rostral middle	186 (0.84)	186.3 (0.73)	0.089 (0.0519)	0.09	0.27	186.4 (0.83)	186.8 (0.76)	0.0888 (0.0658)	0.18	0.60
	Caudal middle	187 (0.7)	187 (0.61)	-0.0173 (0.0596)	0.77	0.85	187.6 (0.69)	187.7 (0.58)	0.0198 (0.0729)	0.79	0.92
	Pars opercularis	186.4 (0.78)	186.6 (0.76)	0.0079 (0.0555)	0.89	0.89	186.9 (0.79)	187.1 (0.82)	0.0364 (0.069)	0.60	0.92
	Pars triangularis	187.4 (0.74)	187.6 (0.64)	0.0174 (0.0604)	0.77	0.85	187.7 (0.75)	188 (0.66)	0.0549 (0.071)	0.44	0.89
	Pars orbitalis	184.7 (0.77)	185 (0.69)	0.0583 (0.0738)	0.43	0.76	185 (0.77)	185.3 (0.69)	0.08 (0.0871)	0.36	0.89
	Lateral orbitofrontal	184.9 (0.84)	185.1 (0.91)	0.0733 (0.0531)	0.17	0.37	185.2 (0.88)	185.2 (1.05)	0.0376 (0.0653)	0.57	0.92
	Medial orbitofrontal	183.2 (0.79)	183.4 (0.86)	0.1479 (0.0612)	0.02	0.12	183.4 (0.82)	183.5 (1.02)	0.1574 (0.0736)	0.03	0.36
	Precentral	189.9 (0.71)	190 (0.64)	-0.0132 (0.0647)	0.84	0.86	190.7 (0.72)	190.8 (0.62)	-0.0033 (0.086)	0.97	0.97
	Paracentral	190.4 (0.74)	190.2 (0.83)	-0.0602 (0.072)	0.40	0.76	191.1 (0.78)	190.8 (0.97)	-0.1765 (0.0893)	0.05	0.36
	Frontal pole	183.2 (0.72)	183.6 (0.6)	0.1651 (0.1113)	0.14	0.34	183.6 (0.73)	184.3 (0.67)	0.4359 (0.1317)	<0.001	**0.03** [c]
	Rostral anterior	179.8 (0.85)	179.9 (0.92)	0.1389 (0.0669)	0.04	0.19	180.1 (0.91)	180.2 (1.09)	0.1542 (0.083)	0.06	0.36
	Caudal anterior	183.8 (0.78)	183.9 (0.85)	0.0269 (0.0707)	0.70	0.85	184.1 (0.84)	184.1 (1.01)	0.0512 (0.0866)	0.56	0.92
Parietal	Superior parietal	188.6 (0.69)	188.7 (0.68)	0.0407 (0.0668)	0.54	0.80	189.5 (0.68)	189.3 (0.74)	-0.1647 (0.0849)	0.05	0.36
	Inferior parietal	186.5 (0.8)	186.8 (0.77)	0.0672 (0.0549)	0.22	0.44	187.1 (0.8)	187.2 (0.85)	-0.0579 (0.069)	0.40	0.89
	Supramarginal	186.9 (0.79)	187.3 (0.74)	0.0924 (0.0592)	0.12	0.31	187.5 (0.78)	187.8 (0.76)	0.0275 (0.0723)	0.70	0.92
	Postcentral	189.4 (0.78)	189.6 (0.72)	-0.0235 (0.0667)	0.72	0.85	190.1 (0.76)	190.2 (0.68)	-0.0976 (0.088)	0.27	0.76
	Precuneus	186.6 (0.82)	186.5 (0.95)	0.0328 (0.0572)	0.57	0.80	187.2 (0.85)	187 (1.1)	-0.1067 (0.0737)	0.15	0.60
	Posterior cingulate	185.1 (0.78)	185.1 (0.84)	-0.0454 (0.06)	0.45	0.76	185.6 (0.82)	185.5 (1.02)	-0.0037 (0.07)	0.96	0.97
	Isthmus cingulate	189.7 (0.77)	189.7 (0.89)	0.0426 (0.0652)	0.51	0.79	190.4 (0.8)	190.3 (1)	0.0069 (0.0806)	0.93	0.97
Temporal	Superior temporal	188.9 (0.73)	189.2 (0.67)	0.0849 (0.0519)	0.10	0.29	189.2 (0.73)	189.5 (0.68)	0.0758 (0.0637)	0.23	0.72
	Inferior temporal	186.7 (0.7)	187.1 (0.6)	0.1163 (0.0534)	0.03	0.17	187 (0.68)	187.4 (0.57)	0.0458 (0.0636)	0.47	0.89
	Middle temporal	187.1 (0.84)	187.6 (0.64)	0.1288 (0.0539)	0.02	0.12	187.4 (0.82)	188.1 (0.56)	0.1639 (0.0694)	0.02	0.31
	Banks of superior temporal sulcus	188.1 (0.78)	188.3 (0.79)	0.112 (0.0608)	0.07	0.23	188.5 (0.82)	188.6 (0.92)	0.0592 (0.0736)	0.42	0.89

© 2025 Puga TB et al. *JAMA Network Open.*

	Fusiform	188.5 (0.84)	188.6 (0.94)	0.0369 (0.0527)	0.48	0.78	188.8 (0.87)	188.8 (1.07)	0.0199 (0.0635)	0.75	0.92
	Transverse temporal	189.1 (0.77)	189.3 (0.81)	0.1145 (0.0847)	0.18	0.37	189.6 (0.81)	189.6 (0.94)	0.0358 (0.1095)	0.74	0.92
	Entorhinal	190.8 (1.01)	190.8 (1.16)	0.0493 (0.0984)	0.62	0.81	190.8 (1.01)	190.6 (1.27)	-0.1564 (0.1137)	0.17	0.60
	Temporal pole	185.5 (0.73)	185.8 (0.77)	0.1838 (0.1006)	0.068	0.23	185.6 (0.68)	185.7 (0.8)	0.086 (0.12)	0.47	0.89
	Parahippocampal	188.9 (0.86)	188.9 (0.99)	0.0334 (0.0653)	0.61	0.81	189 (0.88)	189 (1.12)	-0.0025 (0.0777)	0.97	0.97
Occipital	Lateral occipital	190.5 (0.64)	190.8 (0.52)	-0.0183 (0.0731)	0.80	0.85	190.9 (0.62)	191.1 (0.46)	-0.1513 (0.0914)	0.10	0.48
	Lingual	187.5 (0.82)	187.7 (0.9)	0.2004 (0.0606)	0.001	**0.03** [c]	187.7 (0.85)	187.7 (1.05)	0.0273 (0.0751)	0.72	0.92
	Cuneus	186.4 (0.84)	186.7 (0.91)	0.2403 (0.0763)	0.002	**0.03** [c]	186.8 (0.9)	186.8 (1.07)	0.0393 (0.0962)	0.68	0.92
	Pericalcarine	184.8 (0.84)	185.1 (0.88)	0.2348 (0.084)	0.005	0.06	185 (0.88)	185.1 (1.04)	0.0279 (0.1021)	0.78	0.92
Insula Cortex	Insula	181.9 (0.76)	182.2 (0.8)	0.1049 (0.0571)	0.07	0.23	182.3 (0.8)	182.5 (0.93)	0.0334 (0.0723)	0.64	0.92

[a]: Average T1 intensity of white matter voxels 0.2 mm from the white matter surface for APARC ROI.
[b]: The dependent variables were white matter intensity measures in 34 regions of interest. The independent variable was MTDP exposure. The analysis was adjusted by covariates, including age, sex, race/ethnicity, intracranial volume, pubertal stage, substance ever use, tobacco ever use, parental monitoring, school environment, handedness, imaging device manufacturer, and study site. Sampling weights were incorporated to remove the sampling bias. Regression coefficients (B) measured the difference in white matter intensity variables by MTDP exposure.
[c]: FDR correction was performed across 34 regions to prevent inflation of Type I errors. Footnote indicates FDR<0.05 (bold).

© 2025 Puga TB et al. *JAMA Network Open.*

eReferences

1. Gennatas ED, Avants BB, Wolf DH, et al. Age-Related Effects and Sex Differences in Gray Matter Density, Volume, Mass, and Cortical Thickness from Childhood to Young Adulthood. *J Neurosci*. May 17 2017;37(20):5065-5073. doi:10.1523/JNEUROSCI.3550-16.2017
2. Taki Y, Kawashima R. Brain development in childhood. *Open Neuroimag J*. 2012;6:103-10. doi:10.2174/1874440001206010103
3. Grydeland H, Walhovd KB, Tamnes CK, Westlye LT, Fjell AM. Intracortical myelin links with performance variability across the human lifespan: results from T1-and T2-weighted MRI myelin mapping and diffusion tensor imaging. *Journal of Neuroscience*. 2013;33(47):18618-18630.
4. Norbom LB, Doan NT, Alnæs D, et al. Probing brain developmental patterns of myelination and associations with psychopathology in youths using gray/white matter contrast. *Biological psychiatry*. 2019;85(5):389-398.
5. Reiss AL, Abrams MT, Singer HS, Ross JL, Denckla MB. Brain development, gender and IQ in children. A volumetric imaging study. *Brain*. Oct 1996;119 (Pt 5):1763-74. doi:10.1093/brain/119.5.1763
6. Konrad K, Firk C, Uhlhaas PJ. Brain development during adolescence: neuroscientific insights into this developmental period. *Dtsch Arztebl Int*. Jun 2013;110(25):425-31. doi:10.3238/arztebl.2013.0425
7. Dai HD, Doucet GE, Wang Y, et al. Longitudinal Assessments of Neurocognitive Performance and Brain Structure Associated With Initiation of Tobacco Use in Children, 2016 to 2021. *JAMA Netw Open*. Aug 1 2022;5(8):e2225991. doi:10.1001/jamanetworkopen.2022.25991
8. Modabbernia A, Reichenberg A, Ing A, et al. Linked patterns of biological and environmental covariation with brain structure in adolescence: a population-based longitudinal study. *Molecular Psychiatry*. 2021;26(9):4905-4918.
9. Modabbernia A, Janiri D, Doucet GE, Reichenberg A, Frangou S. Multivariate Patterns of Brain-Behavior-Environment Associations in the Adolescent Brain and Cognitive Development Study. *Biol Psychiatry*. Mar 1 2021;89(5):510-520. doi:10.1016/j.biopsych.2020.08.014
10. Dima D, Modabbernia A, Papachristou E, et al. Subcortical volumes across the lifespan: Data from 18,605 healthy individuals aged 3–90 years. *Human brain mapping*. 2022;43(1):452-469.
11. Herting MM, Johnson C, Mills KL, et al. Development of subcortical volumes across adolescence in males and females: A multisample study of longitudinal changes. *NeuroImage*. 2018;172:194-205.
12. Bernanke J, Luna A, Chang L, Bruno E, Dworkin J, Posner J. Structural brain measures among children with and without ADHD in the Adolescent Brain and Cognitive Development Study cohort: a cross-sectional US population-based study. *The Lancet Psychiatry*. 2022;9(3):222-231.
13. Harrewijn A, Cardinale EM, Groenewold NA, et al. Cortical and subcortical brain structure in generalized anxiety disorder: findings from 28 research sites in the ENIGMA-Anxiety Working Group. *Translational psychiatry*. 2021;11(1):502.

© 2025 Puga TB et al. *JAMA Network Open.*

14. Casey BJ, Cannonier T, Conley MI, et al. The Adolescent Brain Cognitive Development (ABCD) study: Imaging acquisition across 21 sites. *Dev Cogn Neurosci*. Aug 2018;32:43-54. doi:10.1016/j.dcn.2018.03.001
15. Desikan RS, Segonne F, Fischl B, et al. An automated labeling system for subdividing the human cerebral cortex on MRI scans into gyral based regions of interest. *Neuroimage*. Jul 1 2006;31(3):968-80. doi:10.1016/j.neuroimage.2006.01.021
16. Tamnes CK, Herting MM, Goddings A-L, et al. Development of the cerebral cortex across adolescence: a multisample study of inter-related longitudinal changes in cortical volume, surface area, and thickness. *Journal of Neuroscience*. 2017;37(12):3402-3412.
17. Hagler DJ, Jr., Hatton S, Cornejo MD, et al. Image processing and analysis methods for the Adolescent Brain Cognitive Development Study. *Neuroimage*. Nov 15 2019;202:116091. doi:10.1016/j.neuroimage.2019.116091
18. Jefferson AL, Gifford KA, Damon S, et al. Gray & white matter tissue contrast differentiates Mild Cognitive Impairment converters from non-converters. *Brain imaging and behavior*. 2015;9:141-148.
19. Vidal-Ribas P, Janiri D, Doucet GE, et al. Multimodal neuroimaging of suicidal thoughts and behaviors in a US population-based sample of school-age children. *American Journal of Psychiatry*. 2021;178(4):321-332.
20. Puga TB, Dai HD, Wang Y, Theye E. Maternal Tobacco Use During Pregnancy and Child Neurocognitive Development. *JAMA Network Open*. 2024;7(2):e2355952-e2355952.

© 2025 Puga TB et al. *JAMA Network Open.*
