# Supplementary material for: Prenatal Tobacco Exposure, Brain Subcortical Volumes, and Gray-White Matter Contrast
Source: JAMA Netw Open. 2024 Dec 19;7(12):e2451786. doi: 10.1001/jamanetworkopen.2024.51786 (PMC11659912; doi:10.1001/jamanetworkopen.2024.51786)
Supplement: Supplement 2. — Data Sharing Statement [file jamanetwopen-e2451786-s002.pdf]

Data Sharing Statement

Puga. Prenatal Tobacco Exposure, Brain Subcortical Volumes, and Gray-White Matter Contrast. *JAMA Netw Open*. Published December 19, 2024.
doi:10.1001/jamanetworkopen.2024.51786

Data
Data available: Yes
Data types: Deidentified participant data
How to access data: The ABCD data is accessible at https://abcdstudy.org/scientists/data-sharing/.
When available: With publication

Supporting Documents
Document types: None

Additional Information
Who can access the data: The ABCD data is accessible at https://abcdstudy.org/scientists/data-sharing/.
Types of analyses: The ABCD data is accessible at https://abcdstudy.org/scientists/data-sharing/.
Mechanisms of data availability: The ABCD data is accessible at https://abcdstudy.org/scientists/data-sharing/.
